# Hedgehog Inhibitors in Rhabdomyosarcoma: A Comparison of Four Compounds and Responsiveness of Four Cell Lines

**DOI:** 10.3389/fonc.2015.00130

**Published:** 2015-06-08

**Authors:** Rosalie Ridzewski, Diana Rettberg, Kai Dittmann, Nicole Cuvelier, Simone Fulda, Heidi Hahn

**Affiliations:** ^1^Institute of Human Genetics, University Medical Center Goettingen, Goettingen, Germany; ^2^Institute for Cellular and Molecular Immunology, University Medical Center Goettingen, Goettingen, Germany; ^3^Institute for Experimental Cancer Research in Pediatrics, Goethe-University Frankfurt, Frankfurt, Germany

**Keywords:** rhabdomyosarcoma, GDC-0449, LDE225, HhA, cyclopamine

## Abstract

Rhabdomyosarcoma (RMS) is the most common soft tissue sarcoma in children and is divided into two major histological subgroups, i.e., embryonal (ERMS) and alveolar RMS (ARMS). RMS can show HEDGEHOG/SMOOTHENED (HH/SMO) signaling activity and several clinical trials using HH inhibitors for therapy of RMS have been launched. We here compared the antitumoral effects of the SMO inhibitors GDC-0449, LDE225, HhA, and cyclopamine in two ERMS (RD, RUCH-2) and two ARMS (RMS-13, Rh41) cell lines. Our data show that the antitumoral effects of these SMO inhibitors are highly divers and do not necessarily correlate with inhibition of HH signaling. In addition, the responsiveness of the RMS cell lines to the drugs is highly heterogeneous. Whereas some SMO inhibitors (i.e., LDE225 and HhA) induce strong proapoptotic and antiproliferative effects in some RMS cell lines, others paradoxically induce cellular proliferation at certain concentrations (e.g., 10 μM GDC-0449 or 5 μM cyclopamine in RUCH-2 and Rh41 cells) or can increase HH signaling activity as judged by *GLI1* expression (i.e., LDE225, HhA, and cyclopamine). Similarly, some drugs (e.g., HhA) inhibit PI3K/AKT signaling or induce autophagy (e.g., LDE225) in some cell lines, whereas others cannot (e.g., GDC-0449). In addition, the effects of SMO inhibitors are concentration-dependent (e.g., 1 and 10 μM GDC-0449 decrease *GLI1* expression in RD cells whereas 30 μM GDC-0449 does not). Together these data show that some SMO inhibitors can induce strong antitumoral effects in some, but not all, RMS cell lines. Due to the highly heterogeneous response, we propose to conduct thorough pretesting of SMO inhibitors in patient-derived short-term RMS cultures or patient-derived xenograft mouse models before applying these drugs to RMS patients.

## Introduction

Of the major rhabdomyosarcoma (RMS) subtypes in children, embryonal RMS (ERMS) accounts for approximately two-third of cases. It frequently shows loss of heterozygosity of 11p15.5 and overexpression of IGF2. The other subtype is alveolar RMS (ARMS). Seventy-five percent of ARMS harbor reciprocal chromosomal translocations resulting in fusion genes of *PAX3* or *PAX7* and of the forkhead transcription factor, *FOXO1*. Twenty-five percent of ARMS are fusion-negative as are all ERMS. ARMS has a very poor prognosis especially when metastatic (<20% long-term survival) (1). Although the majority of ERMS patients experience more favorable outcomes, treatment failure and toxicity remain substantial. Furthermore, long-term survival of patients with metastatic ERMS is still merely 40% (1). The improvement of treatment schemes is therefore important not only in the combat against ARMS but also against ERMS. This can be only assured if we increase our current knowledge of the basic biology of RMS.

We and others recently showed that predominantly ERMS and fusion-negative ARMS are characterized by high HEDGEHOG (HH) signaling activity (2, 3). Thus, the major marker genes of active HH signaling *GLI1* and *PATCHED1* (*PTCH*) are expressed at significantly higher levels in both subgroups compared to fusion-positive ARMS that however also show activation of the pathway. These results implicate that RMS might be sensitive toward a targeted therapy using small molecule inhibitors directed against components of the HH signaling pathway.

The HH signaling pathway plays a critical role in development, cell fate decisions, and tissue growth. Components of the canonical HH signaling cascade are the HH ligand, the transmembrane protein PTCH that acts as a receptor for HH proteins, the seven-pass transmembrane protein and signaling partner of PTCH, SMOOTHENED (SMO), and the family of GLI transcription factors. In the absence of HH, PTCH inhibits SMO. Binding of HH to PTCH suspends this inhibition. Through a series of poorly understood events that involves SMO-trafficking to the primary cilia, SMO activates the transcription factors GLI2 and GLI3 and the expression of downstream target genes [reviewed in Ref. (4)]. The HH signal also induces the expression of GLI1, which thus amplifies the HH signal. Another downstream target of the pathway is PTCH, which in contrast regulates HH signaling in a negative feedback loop. Together *GLI1* and *PTCH* mRNAs are considered as reliable markers for the pathway’s activity [reviewed in Ref. (5)]. Finally, also *IGF2* expression can be regulated by HH signaling (6, 7). Gene expression data revealed that IGF2 is frequently overexpressed in ERMS and ARMS and plays also a key role in the formation, proliferation, growth, and metastasis of RMS [reviewed in Ref. (8)].

Several drugs targeting the HH pathway exist that already have entered clinical phase I/II trials. The first inhibitor of the HH pathway discovered was cyclopamine. Cyclopamine is a natural product that can be isolated from corn lilies and that binds and inhibits SMO (9). However, because of its limited potency and poor oral solubility, it is not suitable for clinical development (10). Recently, more potent derivatives of cyclopamine and small molecule antagonists targeting SMO have been identified. One of them, i.e., GDC-0449 (vismodegib) has already been approved for advanced basal cell carcinoma (11) and is also used in clinical trials for adult RMS patients. Another compound is LDE225 (sonidegib), which is well-tolerated by the patients and which is currently evaluated in phase II clinical trials for medulloblastoma and RMS. LDE225 also just met primary endpoint in a trial for patients with advanced basal cell carcinoma. In addition, several other compounds, such as BMS-833923 or PF04449913, are being investigated in a range of advanced cancers (10). Table S1 in Supplementary Material is providing a short overview of the four SMO inhibitors used in this study.

SMOOTHENED inhibitors can vary in their capacity to block canonical HH signaling as estimated by *GLI1* expression. They also can have variable potency in blocking the activity of SMO mutational activating variants. Examples are GDC-0449 and HhAntag (HhA). Whereas GDC-0449 has a robust potency against the SMO-E518K variant, but is weakly active against SMOD473H, HhA is essentially equipotent against all SMO alleles (12). Interestingly, besides inhibiting canonical HH signaling, several SMO inhibitors including cyclopamine and GDC-0449 activate a non-canonical SMO/Ca2^+^/AMPK-dependent signaling cascade that may induce a Warburg-like effect, whereas other SMO modulators such as LDE225 do not (13). Together, these data show that SMO inhibitors differ substantially in their mode of action.

Here, we compared the effects of GDC-0449, LDE225, HhA, and cyclopamine with respect to HH pathway inhibition and their potential to inhibit proliferation, to induce apoptosis and to modulate the differentiation status of four different RMS cell lines. We also compared their impact on the activity of other signaling molecules including AKT and AMPK. Our data reveal that the compounds differ extraordinarily in modulation of the above-mentioned parameters and that the responsiveness of the RMS cell lines is highly heterogeneous.

## Materials and Methods

### Reagents

HhA was from Genentech (San Francisco, CA, USA), GDC-0449 (Vismodegib) from Selleckchem (Munich, Germany) and LDE225 (NVP-LDE225) from Active Biochem (Bonn, Germany). For *in vitro* assays, the drugs were dissolved in DMSO. Cyclopamine was purchased from Sigma-Aldrich (St. Louis, MO, USA) and was dissolved in Ethanol. All compounds were easy to solubilize in the respective solvents. For proliferation assays, Annexin V/PI staining and Western blot the final DMSO/ethanol concentrations were uniform in all samples, i.e., the medium contained 0.03% (*v*/*v*) DMSO and 0.1% (*v*/*v*) ethanol, whereas for gene expression analysis and WST-1 assay the solvent was DMSO for GDC-0449, LDE225, and HhA and ethanol for cyclopamine. The final drug concentrations used for *in vitro* analysis are indicated in the respective experiments.

### Cell culture

The human ERMS cell lines RD and RUCH-2 and the ARMS cell lines RMS-13 (also known as Rh30) and Rh41 (also called Rh4) were obtained from ATCC [for cell lines see Ref. (14)]. The ERMS and ARMS cell lines were cultured in DMEM and in RPMI, respectively, 10% FCS (20% FCS for Rh41), and 1% penicillin/streptomycin.

For determination of apoptosis, 10^5^ cells/well (ERMS) or 15 × 10^4^ cells/well (ARMS) were seeded in six-well-plates. After treatment for 48 h with medium supplemented with drugs or solvent as indicated in the respective experiments, apoptosis was determined by flow cytometry on a FACSCalibur (BD Biosciences, Heidelberg, Germany) after staining of the cells with Annexin V-FITC (BD Biosciences, Heidelberg, Germany) and Propidium Iodide (PI, Miltenyi Biotec, Bergisch Gladbach, Germany).

For BrdU incorporation and WST assay, 4000 cells/well were seeded in 96-well-plates. For the BrdU assay, cells were incubated for 24 h with the respective drugs in the presence of BrdU. BrdU incorporation was measured using a Cell Proliferation BrdU ELISA (Roche Diagnostics GmbH, Mannheim, Germany). The data are presented as the percentage of the incorporation measured in time-matched solvent-treated controls taken as 100%. For WST-1 assay, cells were incubated for 24 h with the respective drugs. Four hours before the end of incubation, WST-1 reagent (Roche Diagnostics, Mannheim, Germany) was added as recommended by the manufacturer. The amount of produced formazan dye (quantitated by spectrophotometer at a wavelength of 450 nm) correlates to metabolic active cells.

Data shown summarize three independent experiments performed as duplicates (apoptosis assay) or triplicates (BrdU incorporation assay) or two independent experiments performed as triplicates (WST-1 assay).

### Real-time quantitative RT–PCR-analyses

For gene expression analysis, 10^5^ cells/well (ERMS) or 15 × 10^4^ cells/well (ARMS) were seeded in six-well-plates. After incubation of the cells for 24 h, total RNA was isolated using TRIzol Reagent (Invitrogen GmbH, Karlsruhe, Germany) and cDNA was synthesized using Superscript II and random hexamers (Invitrogen, Karlsruhe, Germany). Quantitative RT–PCR of target cDNAs was performed using SYBR-green based assays on an ABI Prism HT 7900 Detection System instrument and software (Applied Biosystems, Darmstadt, Germany). The primers for amplification of target transcripts are shown in the Table S2 in Supplementary Material. All primers used in study were intron-flanking, except of the primers for *18S* and *hMYOD*. Expression levels of *18S rRNA* served to normalize the transcript levels. Each sample was measured in triplicates. Expression of major components of the HH signaling pathway was analyzed once. All other data shown are the summary of two independent experiments performed in duplicates. Graphs represent the mean value of all measurements plus SEM.

### Western blot analysis

Preparation of cell lysates and determination of protein concentrations were done as described previously (15). Primary antibodies used to detect the individual target proteins and corresponding secondary antibodies are shown in Table S3 in Supplementary Material. All Western blots shown are representative for at least two independent experiments.

### Statistical analysis

When comparing two samples, statistical differences were analyzed using Student’s *t*-test. Data were considered significant when *p* < 0.05.

## Results

### Effects of GDC-0449, LDE225, HhA, and cyclopamine on HH signaling activity and on the expression of *IGF2* and of muscle differentiation markers

We first examined whether the two ERMS cell lines, RD and RUCH-2, and the two ARMS cell lines, RMS-13 and Rh41, used in the study express the major components of the HH signaling cascade *SHH*, *SMO*, *PTCH*, and *GLI1-3*. Of these genes, *GLI1* and *PTCH* are regulated by the HH signal and thus are HH pathway target genes. As shown in Figure S1 in Supplementary Material, *SHH*, *SMO*, and *PTCH* and the transcriptional effectors *GLI1*, *GLI2*, and *GLI3* were expressed in all cell lines. Expression of *GLI1*, *GLI3*, *PTCH*, and *SMO* was highest in RMS-13 cells that show an amplification of *GLI1* (16). Furthermore, we detected *SHH* expression that was highest in RUCH-2 cells. In summary, all RMS cell lines express the major components of the HH signaling pathway. This is similar to data previously published by Graab et al. (17).

Because *GLI1* and *PTCH* expression were suggestive for canonical HH signaling activity (i.e., via the HH/PTCH/SMO/GLI axis) in the different cell lines, we examined whether it is possible to inhibit HH signaling using the SMO inhibitors GDC-0449, LDE225, HhA, and cyclopamine. For this purpose, we first determined the optimal conditions under which each inhibitor may block HH signaling. In order to measure HH signaling activity, the transcription of the HH pathway indicator *GLI1* was analyzed by qRT-PCR.

**Figure 1:**
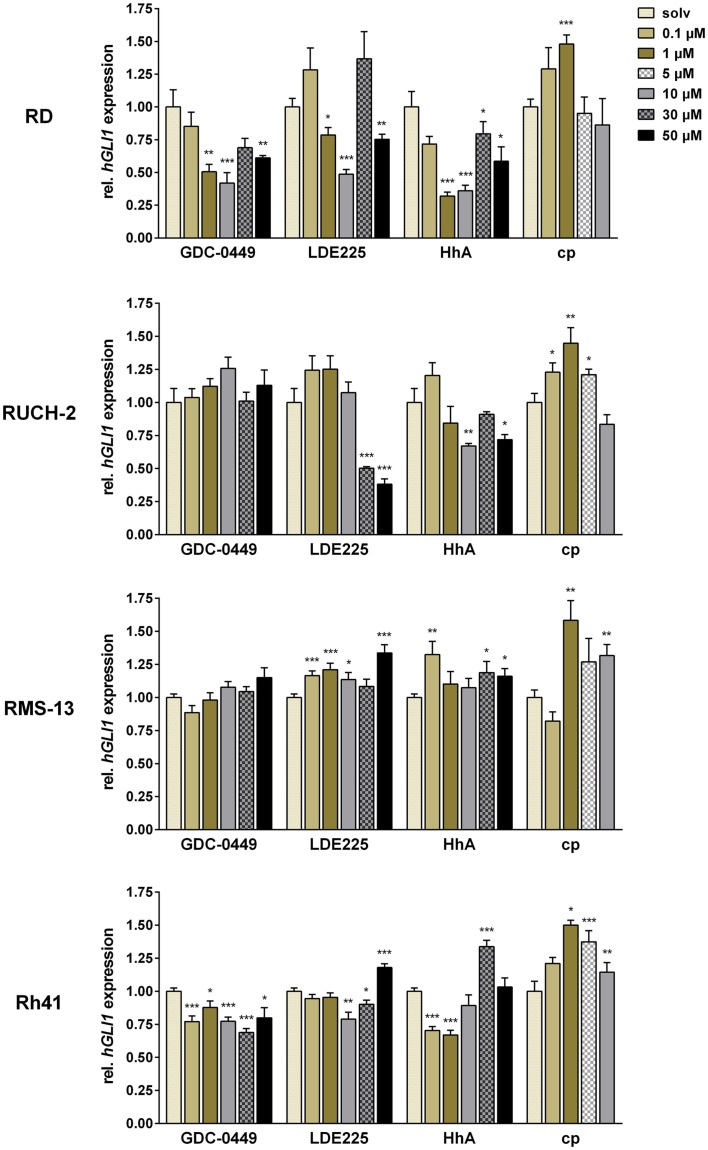
**Relative quantification of *GLI1* expression in RD, RUCH-2, RMS-13, and Rh41 after treatment with GDC-0449, LDE225, HhA, and cyclopamine (cp) at the concentration indicated**. Gene expression levels were normalized to *18S* rRNA expression levels. The respective solvent-controls (solv) for each experiment were set to 1. All data represent at least two independent experiments performed in duplicates and measured in triplicates. The data are represented as mean ± SEM; *p < 0.05; **p < 0.01; ***p < 0.001.

In the cell line RD, significant inhibition of *GLI1* expression was revealed for 1, 10, and 50 μM GDC-0449 and LDE225, and for 1–50 μM HhA (Figure 1). Interestingly, we found that 30 μM of GDC-0449 or LDE225 did not further decrease HH signaling activity but restored the activity to basal level or above (Figure 1). Similarly, cyclopamine increased *GLI1* expression and the increase was significant at 1 μM (Figure 1).

In RUCH-2 cells, the dose-response analyses revealed that GDC-0449 did not significantly modulate *GLI1* expression at any concentration, whereas LDE225 and HhA inhibited *GLI1* expression at 30 and 50 μM, and 10 and 50 μM, respectively. In addition, and similar to RD cells, cyclopamine increased *GLI1* expression at concentrations of 0.1, 1, and 5 μM (Figure 1).

In RMS-13 cells, which show *GLI1* amplification, the drugs did not decrease *GLI1* expression levels at any concentrations used in the experiments. Rather *GLI1* expression was increased, which was particularly obvious for LDE225 and cyclopamine (Figure 1).

In Rh41, we found that GDC-0449 moderately inhibited *GLI1* expression at any concentration whereas LDE225 inhibited *GLI1* expression only at 10 and 30 μM, and increased it at 50 μM. HhA inhibited *GLI1* expression at a concentration of 0.1 and 1 μM, whereas higher concentrations also resulted in restoration or increase in *GLI1* expression. Cyclopamine never inhibited *GLI1* expression but induced it for 1–10 μM (Figure 1).

Together, these data show that ERMS and ARMS cell lines show tumor-intrinsic HH signaling activity as estimated by modulation of *GLI1* expression after treatment with SMO – antagonists. Furthermore, our data demonstrate that SMO inhibitors – dependent on the concentration – may paradoxically activate *GLI1* transcription in some RMS cell lines.

We next measured the expression of *IGF2* that plays an important role in RMS pathology (8) and is regulated by HH signaling in specific cellular settings (6, 7). Whereas RUCH-2 cells did not express *IGF2*, the levels of *IGF2* were regulated by all drugs in the remaining three cell lines, however to a variable extent (Figure S2 in Supplementary Material). In RD cells, *IGF2* transcription was inhibited by 0.1, 30, and 50 μM GDC-0449, by 50 μM LDE225 and by 10, 30, and 50 μM HhA. Cyclopamine had no effect and 0.1 μM HhA increased the *IGF2* levels. In RMS-13, *IGF2* expression was significantly inhibited upon treatment with 50 μM HhA or LDE225 and with 0.1 and 1 μM GDC-0449. By contrast, 0.1 and 1 μM HhA as well as 1–10 μM cyclopamine resulted in a significant up-regulation of the expression of this gene. This was different in Rh41 cells. In this cell line, the *IGF2* mRNA level was significantly decreased only with 10 μM cyclopamine, whereas all other drugs rather increased it. This was specifically obvious after GDC-0449-treatment (Figure S2 in Supplementary Material).

Finally, we also examined the expression of the early and late muscle differentiation markers *MYOD* and *MYH1*, respectively (Figure S3 and S4 in Supplementary Material).

RUCH-2 cells neither expressed *MYOD* nor *MYH1*.

In RD cells, *MYOD* transcription was inhibited by 0.1 and 50 μM GDC-0449, whereas 1 μM GDC-0449 induced it. *MYOD* expression was also inhibited by 0.1, 10, and 50 μM LDE225, by 10, 30, and 50 μM HhA and 0.1 and 1 μM cyclopamine. In RMS-13, *MYOD* expression was significantly inhibited upon treatment with 1 and 30 μM GDC-0449, 30 and 50 μM LDE225 or HhA and with 0.1 and 1 μM cyclopamine. In Rh41 cells, GDC-0449 and LDE225 induced *MYOD* expression at any concentration. *MYOD* was also induced by 10–50 μM HhA, whereas cyclopamine had no effect.

*MYH1* transcription in RD cells was inhibited by 30 and 50 μM GDC-0449, by 10–50 μM LDE225, by 1–50 μM HhA, whereas it was induced by cyclopamine at any concentration. In RMS-13, *MYH1* expression was induced by GDC-0449 (significant for 0.1 and 30 μM) and by LDE225 (significant for 1–30 μM). HhA induced the expression at a concentration of 0.1 and 1 μM, whereas 30 and 50 μM inhibited it. Inhibition of *MYH1* expression was also seen with 5 μM cyclopamine. In Rh41, *MYH1* expression was induced by GDC-0449 (significant for 0.1, 10, and 30 μM) and by 1 and 30 μM LDE225, whereas 50 μM inhibited it. HhA induced the expression at a concentration of 0.1 and 1 μM, whereas 30 and 50 μM inhibited it. Inhibition of *MYH1* expression was also seen with 1–10 μM cyclopamine.

Together, SMO inhibitors may – dependent on the concentration – activate or inhibit transcription of *IGF2* and that of muscle differentiation markers in some RMS cell lines. Furthermore, the responses of the cell lines are highly variable and differ enormously from each other.

### Effects of GDC-0449, LDE225, HhA, and cyclopamine on cellular proliferation and apoptosis

Next, we assessed the antiproliferative and proapoptotic effects of SMO inhibitors. For this purpose, we used concentrations that either have decreased or did not impact (10 and 30 μM, respectively) on *GLI1* expression in most experimental settings. Cyclopamine was used only at a concentration of 5 μM.

**Figure 2:**
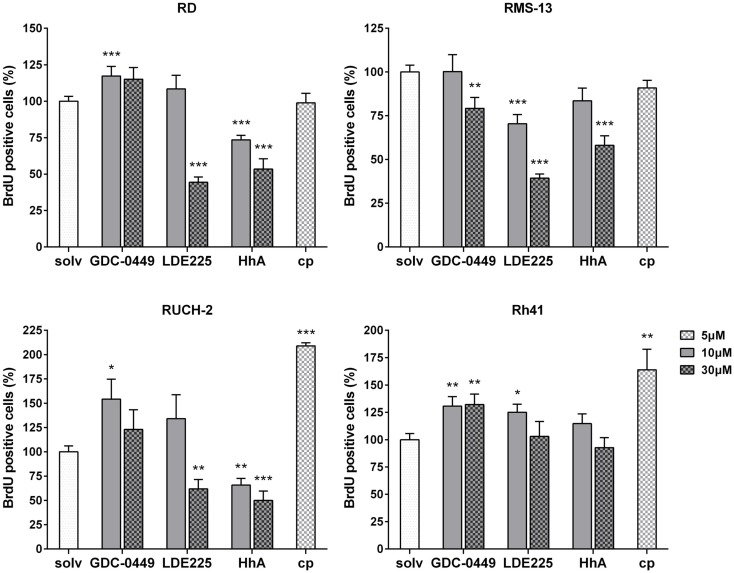
**BrdU incorporation in RD, RUCH-2, RMS-13, and Rh41 cells after incubation with GDC-0449, LDE225, HhA, and cyclopamine (cp) at the concentration indicated**. BrdU-incorporation is shown as percentage of respective solvent-controls (solv) that were set to 100%. The data are represented as mean ± SEM; *p < 0.05; **p < 0.01; ***p < 0.001.

As shown by BrdU assays, the antiproliferative effects of SMO inhibitors dramatically varied between the cell lines: in RD cells, 30 μM LDE225 and 10 μM and 30 μM HhA reduced the proliferation rate of RD cells approximately by 55% and 30 and 50%, respectively (Figure 2). Thirty micromoles of GDC-0449, 10 μM LDE225 and cyclopamine did not show any antiproliferative effects, whereas 10 μM GDC-0449 significantly increased the proliferation rate (Figure 2). This was similar in RUCH-2 cells, in which the proliferation was significantly reduced by 30 μM LDE225 and by 10 and 30 μM HhA (Figure 2). Ten micromoles of LDE225 and 30 μM GDC-0449 had no effects, whereas 10 μM GDC-0449 and 5 μM cyclopamine significantly increased it (by approximately 50 and 100%, respectively) (Figure 2). In RMS-13, proliferation was reduced by 30 μM GDC-0449, by 10 and 30 μM LDE225 and by 30 μM HhA (Figure 2). In Rh41, none of the drugs inhibited proliferation (Figure 2). However, proliferation-inducing effects were seen for 10 and 30 μM GDC-0449, for 10 μM LDE225 and for cyclopamine (Figure 2; for RD and Rh41 the results were confirmed by simple cell counting; see Figure S5 in Supplementary Material).

**Figure 3:**
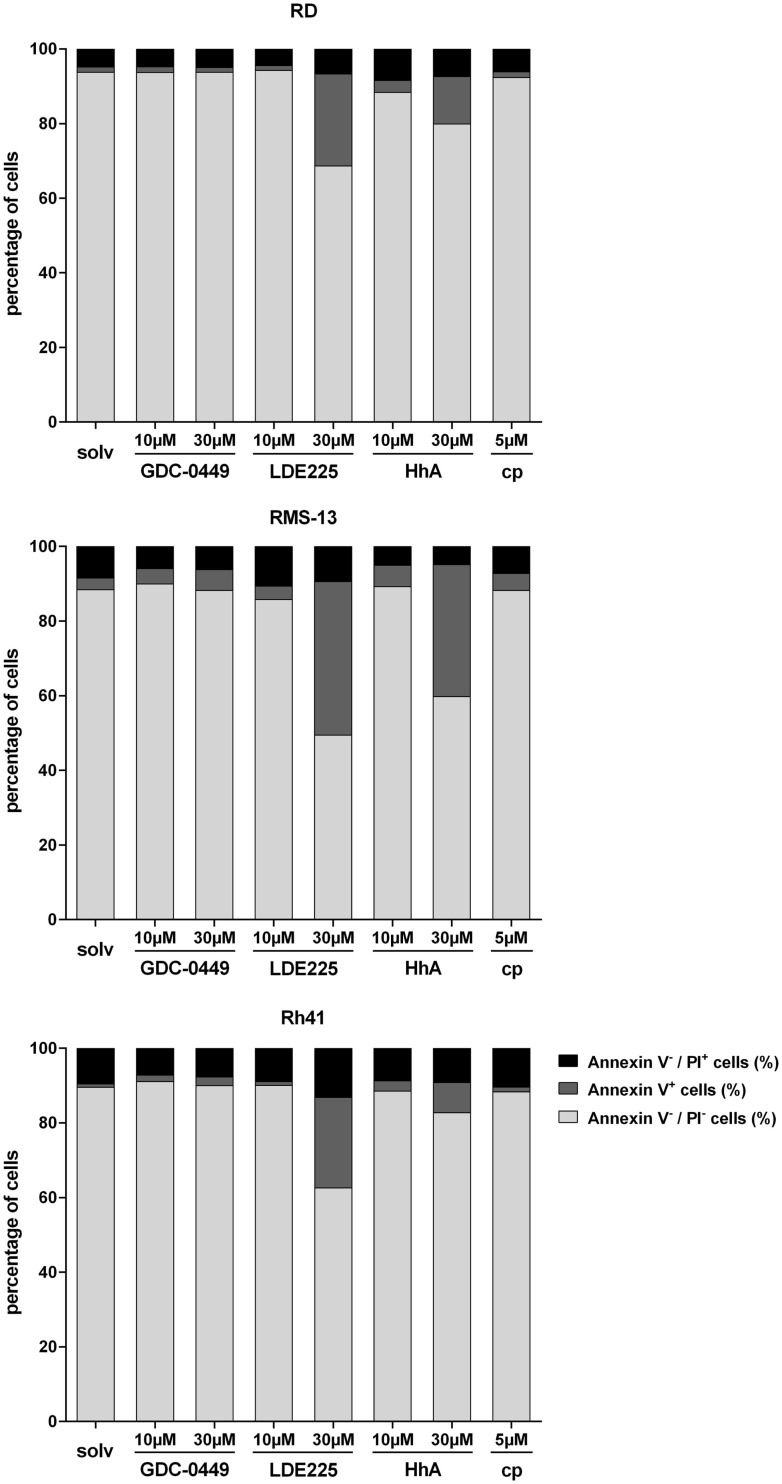
**Analysis of Annexin V/PI positivity of RD, RMS-13 and Rh41 cells after treatment with GDC-0449, LDE225, HhA, and cyclopamine (cp) at the concentration indicated**. Apoptosis was analyzed by FACS after staining of the cells with Annexin V and Propidium Iodide (PI). Vital cells are Annexin V^−^/PI^−^, early-apoptotic (Annexin V^+^/PI^−^) and late-apoptotic cells (Annexin V^+^/PI^+^) were summarized as Annexin V^+^ cells and Annexin V^−^/PI^+^ are necrotic cells, respectively. All data represent at least three independent experiments measured in duplicates. The data are represented as mean ± SEM; solv, solvent.

As far as apoptosis is concerned, 30 μM LDE225 significantly increased the apoptosis rate (as estimated by increase of Annexin V positive cells) of RD cells as did 30 μM HhA (Figure 3). Ten micromoles of LDE225 or HhA as well as GDC-0449 and cyclopamine did not trigger apoptosis (Figure 3). The substances had similar effects in RMS-13 and Rh41 cells (Figure 3). Assessment of apoptosis in RUCH-2 cells was not possible due to unstable results obtained from three measurements.

Together, these data demonstrate that the responsiveness of the cell lines to four distinct SMO inhibitors is very heterogeneous. In general, the results show that cyclopamine at the used concentration neither induces apoptosis nor reduces proliferation of any of the cell lines. It rather seems to increase the proliferation rate in RUCH-2 and Rh41 cells. In addition, GDC-0449 is less effective compared to LDE225 or HhA that have the capacity to induce apoptosis and antiproliferative effects dependent on the cell line and the applied drug concentration.

### Effects of GDC-0449, LDE225, HhA, and cyclopamine on PI3K/AKT/mTOR signaling activity and activation of AMPK and LC3

HEDGEHOG signaling can influence the activation status of PI3K/AKT signaling (18, 19, 20) and can inhibit autophagy (21). In addition, HH inhibitors can impact on a non-canonical SMO/Ca^2+^/AMPK-dependent signaling cascade (13). Therefore, we examined the phosphorylation status of AKT and AMPK. We also measured the phosphorylation status of S6 that can be taken as surrogate readout for mTOR activity, and the activation status of caspase 3. Autophagy was assessed by the expression levels of the cytosolic 16 kDa large LC3-I and the 14 kDa large LC3-II, the latter being the lipidated form of LC3-I and specifically associates with autophagosome membranes and thus reflect autophagosome numbers (22). In the following, the most important and clear-cut findings from at least two independent Western blot analyses are summarized.

**Figure 4:**
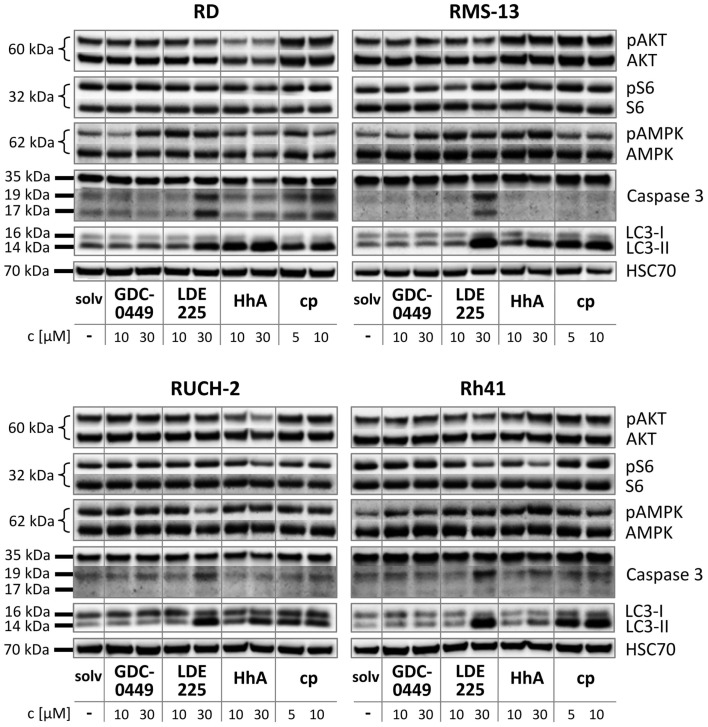
**Western blot analysis of RD, RUCH-2, RMS-13, and Rh41 after treatment with GDC-0449, LDE225, HhA, and cyclopamine (cp) at the concentration indicated. solv, solvent**.

In RD cells, GDC-0449, LDE225, and cyclopamine did not influence the phosphorylation status of AKT or S6 in comparison to the solvent (Figure 4). In contrast, treatment with HhA reduced AKT/pAKT levels in RD cells (Figure 4). An induction of AMPK phosphorylation was seen with 30 μM GDC-0449 and with 10 and 30 μM LDE225 (Figure 4). Caspase 3 activity was induced by 30 μM LDE225 as was autophagy (Figure 4). Increased LC3-II levels were also seen after treatment with HhA and cyclopamine at any concentration (Figure 4).

In RUCH-2 cells and similar to RD cells, HhA reduced AKT/pAKT levels and 30 μM LDE225 induced caspase 3 activity and autophagy (Figure 4). Furthermore, 30 μM LDE225 reduced pAMPK phosphorylation (Figure 4). An increase in LC3-II expression was also detected with HhA and cyclopamine (Figure 4).

In RMS-13 cells, we found a clear-cut effect of 30 μM LDE225 on caspase 3 activation and on LC3-II levels (Figure 4). LC3-II was also increased by 30 μM HhA and by cyclopamine at any concentration (Figure 4).

In Rh41 cells, GDC-0449 and HhA did not exert any obvious effect (Figure 4). LDE225 at a concentration of 30 μM strongly induced caspase 3 activity and increased LC3-II levels (Figure 4). Increase in LC3-II was also detected after treatment of the cells with cyclopamine (Figure 4).

In summary, these data show that the drugs exert highly heterogeneous effects on the cell lines and that the responsiveness of the cell lines is very heterogeneous as well.

## Discussion

The HH pathway controls cell proliferation, differentiation, and tissue patterning. Thus, it is not surprising that a pathological activation of the pathway results in tumor formation. The cancers include those of the skin, brain, prostate, lungs, breast, and also subgroups of RMS. Therefore, the HH pathway is considered a good target for therapy. Several compounds exist and their efficacies are currently tested in clinical studies. One of these drugs is GDC-0449 that has already been approved for advanced and metastasizing basal cell carcinoma (10).

Despite these promising trends, HH inhibitors can rapidly lead to resistance. For example, GDC-0449 resistance has occurred in medulloblastoma through a D473H mutation in SMO, which prevented GDC-0449-SMO binding while maintaining aberrant HH signaling (23, 24). It also has been reported that resistance under LDE225 treatment include amplification of *GLI2*, aberrant up-regulation of PI3K signaling, and *SMO* mutations (25). This indicates that it is important to select those inhibitors for therapy that instantly and very potently inhibit proliferation and/or induce apoptosis of the tumor cells to circumvent the occurrence of these resistance mechanisms.

Our data on RMS show that LDE225 and HhA meet these criteria in RUCH-2, RD, and RMS-13 cell lines at concentrations between 10 and 30 μM, whereas cyclopamine and also GDC-0449 do not. Instead the latter drugs rather *increased* BrdU incorporation and exerted no proapoptotic effects. The *increase* in BrdU incorporation upon cyclopamine and GDC-0449 treatment are hard to explain, especially because it was neither correlated with induction of HH signaling nor IGF2 overexpression. *Vice versa*, the antiproliferative and proapoptotic effects of LDE225 and HhA not necessarily correlated with inhibition of HH signaling. By contrast, we observed that these compounds (and also the other used in the study) had the potency to *increase GLI1* expression levels. This was dependent on the cell line, on the applied drug, and the respective drug concentration. For example, treatment of RMS-13 cells with 30 μM HhA or LDE225 resulted in a strong antiproliferative and proapoptotic effect but in an *up-regulation* of *GLI1*. Curiously, the proapoptotic effects (as measured by Annexin V labeling) were associated with caspase 3 activity in LDE225-treated cells, but not in HhA-treated ones, indicating that HhA may trigger caspase 3-independent apoptosis.

We are not aware of any study showing that SMO inhibitors can *induce GLI1* expression. Although it is possible that this effect is specific for RMS cell lines, it is more likely that the inhibitors influence other signaling molecules that in turn activate *GLI1* expression in a dose-dependent manner. This scenario has been demonstrated for the SMO agonist SAG (26). Thus, Chen and colleagues showed that SAG induced HH signaling activity with an EC50 of 3 nM, but inhibited the activity at concentrations above 1 μM. The authors propose a model, in which SAG may interact not only with SMO, but also with another cellular effector of SMO activation. According to their model, optimal SAG concentrations induce HH pathway activation by facilitating the association of SMO with the respective protein, whereas higher SAG concentrations begin to inhibit this process, as the agonist would independently bind both SMO and the effector (26). Similar models may apply to SMO inhibitors.

It is also possible that the observed concentration-dependent effects of the drugs on *GLI1* expression are off-target effects of the SMO inhibitors. Toxic effects can be rather excluded. A good example are RD cells that show a triphasic *GLI1* expression profile, i.e., a decrease at 10 μM, an increase at 30 μM, and a decrease at 50 μM upon GDC-0449, LDE225, or HhA treatment, but no triphasic changes in metabolic activity that continuously decreases with increasing drug concentration (compare Figure 1 and Figure S6 in Supplementary Material). Thus, an increase of *GLI1* expression (at 30 μM) is not correlated with increased cell viability (Figure S6 in Supplementary Material).

Furthermore, Dijkgraaf and colleagues (12) discussed a two-step mechanism for SMO activation that not only requires the transport to the cilia, but also an unidentified second activation step that allows SMO to trigger downstream HH signaling. The authors suggest that SMO antagonists can be subdivided in two classes. Whereas one class of inhibitors influences trafficking of SMO to cilia, the other class affects the activation step. They also discuss the possibility that SMO antagonists can induce slightly different SMO conformations that favor a particular subcellular localization over another (12). This also could explain some of the different effects of the inhibitors used in our study.

HEDGEHOG signaling also regulates the expression of muscle differentiation markers. MYOD expression has been shown to be inhibited by GLIs (27). In addition, HH signaling can inhibit terminal muscle differentiation (28). We therefore hypothesized that inhibition of HH signaling using SMO antagonists should result in up-regulation of *MYOD* and *MYH1* and thus in a more differentiated RMS phenotype. However, our data show that this only applied to some inhibitors at specific concentrations in specific cells lines (e.g., GDC-0449 at any concentration in Rh41 cells), whereas the genes were rather down-regulated in other cell lines (e.g., by LDE225 in RD cells). This again indicates that the drugs have heterogeneous effects.

It also has been demonstrated that SMO inhibitors (besides inhibiting the canonical SMO-GLI axis) can activate a non-canonical SMO/Ca^2+^/AMPK axis that triggers a rapid Warburg-like catabolic reprograming. Induction of a Warburg-like effect has been shown for cyclopamine and GDC-0449, but not for LDE225, in mature 3T3-L1 adipocytes and the myoblast cell line C2C12 (13). It has been suggested that the physiological effects of these non-canonical endpoints, i.e., Ca^2+^ influx and AMPK-mediated catabolism, are consistent with the two major reported side effects of SMO inhibitors such as muscle cramping and weight loss. Although we did not measure Ca^2+^ influx our data shows that the AMPK phosphorylation was indeed induced in RD cells with 30 μM GDC-0449 and with 10 and 30 μM LDE225. This indicates that also LDE225 can induce Warburg-like effect, which apparently depends on the drug concentration and the cellular context.

Recently, it has been demonstrated that HH signaling can activate PI3K/AKT signaling and that, *vice versa*, AKT can stabilize and thus activate GLI transcription factors (18, 19, 20). In addition, mTOR, which is a downstream target of PI3K/AKT signaling can also trigger GLI activation (29). Therefore, we analyzed whether SMO inhibitors modulate activation of AKT or mTOR in RMS cell lines. Indeed, HhA, but none of the other SMO antagonists, blocked phosphorylation of AKT in both ERMS cell lines. Effects on S6 phosphorylation were not seen. Although this suggests that there is a crosstalk between HH and AKT signaling in at least some RMS, it is possible that HhA induces HH-independent processes that trigger AKT inhibition.

Because HH signaling can inhibit autophagosome synthesis, both in basal and in autophagy-induced conditions (21), we finally investigated whether the applied drugs induced autophagy. Indeed, our data show that HhA, LDE225, and cyclopamine can strongly increase the levels of LC3-II. Induction of autophagy by SMO inhibitors has also been shown in a variety of other cancer cell lines. Examples are hepatocellular and pancreatic carcinoma, in which the GLI inhibitor GANT61 induced autophagy that was accompanied with reduced cell viability and increased apoptosis both *in vivo* and *in vitro* (30, 31). The authors proposed that HH signaling by regulating autophagy plays an important role in determining the cellular response to HH-targeted therapy in pancreatic cancer. However, our data shows that induction of autophagy by HH inhibitors is not necessarily accompanied by antiproliferative or proapoptotic effects. For example, this is demonstrated in Rh41 cells that upon cyclopamine treatment showed increased LC3-II levels, but also increased BrdU incorporation and no alteration in the number of Annexin V positive cells.

Together, our study reveals that the mode of action of the applied HH inhibitors differs substantially from each other. Although HhA and LDE225 are probably the most universal HH inhibitors and exert antitumoral effects in almost all RMS cell lines used in our study, we propose to conduct thorough pretesting of HH inhibitors before applying these drugs to RMS patients. This could be done either in patient-derived short-term RMS cultures or in patient-derived xenograft mouse models.

## Conflict of Interest Statement

The authors declare that the research was conducted in the absence of any commercial or financial relationships that could be construed as a potential conflict of interest.

## Supplementary Material

The Supplementary Material for this article can be found online at http://journal.frontiersin.org/article/10.3389/fonc.2015.00130/abstract
