# Supplementary material for: Hedgehog Inhibitors in Rhabdomyosarcoma: A Comparison of Four Compounds and Responsiveness of Four Cell Lines
Source: Front Oncol. 2015 Jun 8;5:130. doi: 10.3389/fonc.2015.00130 (PMC4459089; doi:10.3389/fonc.2015.00130)
Supplement: Supplementary file 1 [file Datasheet_1.PDF]

Supplementary Material

Hedgehog inhibitors in rhabdomyosarcoma: a comparison of 4 compounds and responsiveness of 4 cell lines

Rosalie Ridzewski[1], Diana Rettberg[1], Kai Dittmann[2], Nicole Cuvelier[1], Simone Fulda[3], Heidi Hahn[1]*

[1] Institute of Human Genetics, University Medical Center Goettingen, Germany
[2] Institute for Cellular and Molecular Immunology, University Medical Center Goettingen, Germany
[3] Institute for Experimental Cancer Research in Pediatrics, Goethe-University Frankfurt, Germany

Running title: Hedgehog inhibitors and rhabdomyosarcoma

Keywords: rhabdomyosarcoma, GDC-0449, LDE225, HhA, cyclopamine

*Corresponding author: Heidi Hahn, Department of Human Genetics, University Medical Center Goettingen, Heinrich-Dueker Weg 12, 37073 Goettingen, Germany. Phone: +49 551 3914010; Fax: +49 551 396580; e-mail: hhahn@gwdg.de

Supplemental Table 1: Characteristics of SMO inhibitors used in the study

Compound	Source	General information	Clinical development	Ref.
**Cyclopamine**	Natural compound	First discovered HH inhibitor; teratogen	Not suitable	[1],[2]
**GDC-0449** (vismodegib)	Curis/ Evotech/ Genentech	approved SMO inhibitor	Approved for advanced basal cell carcinoma in 2012; Clinical trials for adult RMS patients	[3]
**LDE225** (sonidegib)	Novartis	Well tolerated HH inhibitor	Reached primary endpoint in a trial for advanced basal cell carcinoma; Phase II for medulloblastoma and RMS; Currently in clinical development for a variety of diseases	[4]
**HhA** (HhAntag, HhAntag691)	Curis/ Evotech/ Genentech	Precursor of GDC-0449, tool compound to block Hh signaling in mice	Suboptimal as a therapeutic agent	[5]

Note: For chemical structure of the applied SMO inhibitors see [5] and [6] of supplemental references.

Supplemental Table 2: Oligonucleotides used for qRT-PCR

Transcript	Primer name forward/reversed	Primer sequence (5’ – 3’ orientation)	Exon
18S	18S-fwd	CGCAAATTACCCACTCCCG	1
	18S-rev2	TTCCAATTACAGGGCCTCGAA	1
hGLI1	hsaGLI1 tqF	AGCTACATCAACTCCGGCCA	11
	hsaGLI1 tqR	GCTGCGGCGTTCAAGAGA	12
hGLI2	hsGLI2F.1	AAGCCCTTCAAGGCGCAGTA	6
	hsGLI2R.1	TCGTGCTCACACACATATGGCTT	7
hGLI3	hsGLI3F.1	GCCAGCGCAGCCCCTAT	6
	hsGLI3R.1	CGGCCTGGCTGACAGCCT	7
hIGF2	hIGF2 F2	GACACCCTCCAGTTCGTCTG	2
	hIGF2 F1	ATTGGAAGAACTTGCCCACG	4
hMYH1	hsMYH1F.1	TGTGCAGCAGGTGTACAATGC	13, 14
	hsMYH1R.1	TGCACAGCTGCTCCAGGCT	15
hMYOD	hMYOD F	CGAACCCCCAACCCGATA	3
	hMYOD R	GAAAAAACCGCGCTGTGT	3
hPTCH	hsPTC1F.2	GAGGTTGGTCATGGTTACATGGA	6
	hsPTC1R.2	TGCTGTTCTTGACTGTGCCACC	7
hSHH	hSHH_forw	GATGACTCAGAGGTGTAAGGAC	1,2
	hSHH_rev	CCTCGTAGTGCAGAGACTCC	2
hSMO	hSMO-F1	CAAGAACTACCGATACCGTGC	6
	hSMO-R1	AGCATGGTCTCGTTGATCTTGC	7

Supplemental Table 3: Primary and secondary antibodies for Western Blot

Antibody	Product and company	Dilution
**Primary antibody**		
Mouse anti-AKT	mAB, 610861, BD Biosciences Pharmingen	1:1000
Mouse anti-HSC70	mAB, sc-7298, Santa Cruz	1:10000
Mouse anti-S6	mAB, 54D2, Cell Signaling	1:1000
Rabbit anti-AMPK	pAB, Cell Signaling	1:1000
Rabbit anti-ß-Actin	mAB, 13E5, Cell Signaling	1:1000
Rabbit anti-Caspase 3	pAB, Cell Signaling	1:1000
Rabbit anti-IGF2	pAB, LS-C165143, LSBio	1:500
Rabbit anti-LC3B II	mAB, D11 XP, Cell Signaling	
Rabbit anti-pAKT (Ser473)	mAB, 193H12, Cell Signaling	1:1000
Rabbit anti-pAMPK (Thr172)	mAB, 40H9, Cell Signaling	1:1000
Rabbit anti-pS6 (Ser240/244)	pAB, Cell Signaling	1:1000
**Secondary antibody**		
Goat anti-Rabbit/HRP	pAB, A0545, Sigma-Aldrich	1:5000
Sheep anti-mouse/HRP	pAB, NA931, GE Halthcare	1:5000

Supplemental Figure 1: Relative quantification of expression of HH pathway genes in RD, RUCH-2, RMS-13 and Rh41. Gene expression levels of *SHH*, *SMO*, *PTCH* and *GLI1-3* were normalized to *18S* rRNA expression levels. Data represent one experiment performed and measured in triplicates, respectively. The data are represented as mean +/-SEM.

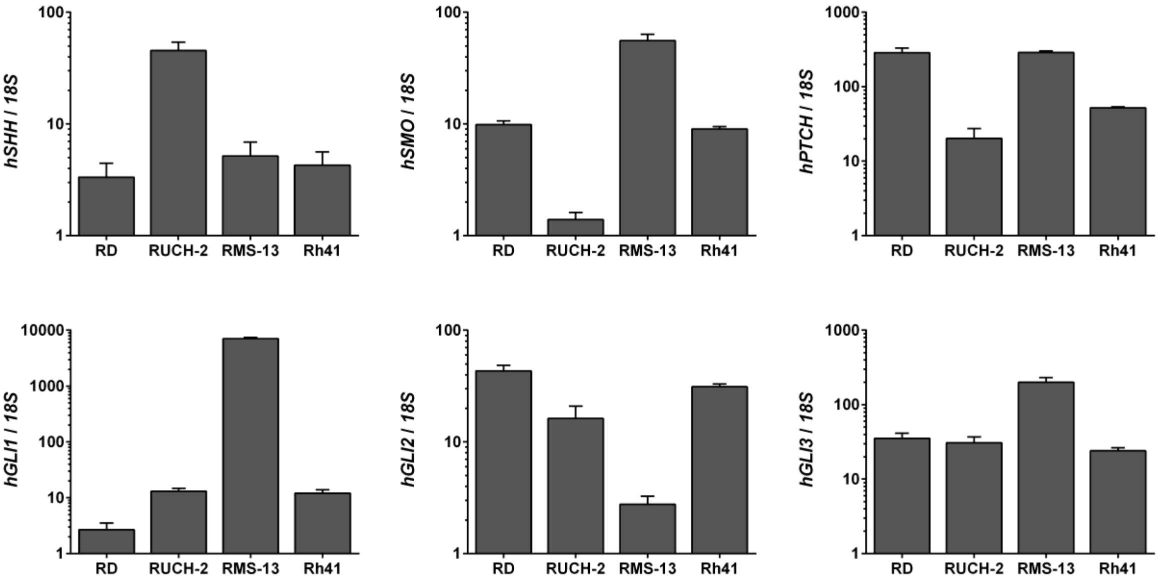

Supplemental Figure 2: Relative quantification of *IGF2* expression in RD, RMS-13 and Rh41 after treatment with GDC-0449, LDE225, HhA and cyclopamine (cp) at the concentration indicated. Gene expression levels were normalized to *18S* rRNA expression levels. The respective solvent-controls (solv) were set to 1. All data represent at least 2 independent experiments performed in duplicates and measured in triplicates. The data are represented as mean +/-SEM; *, p<0.05; **, p<0.01; ***, p<0.001.

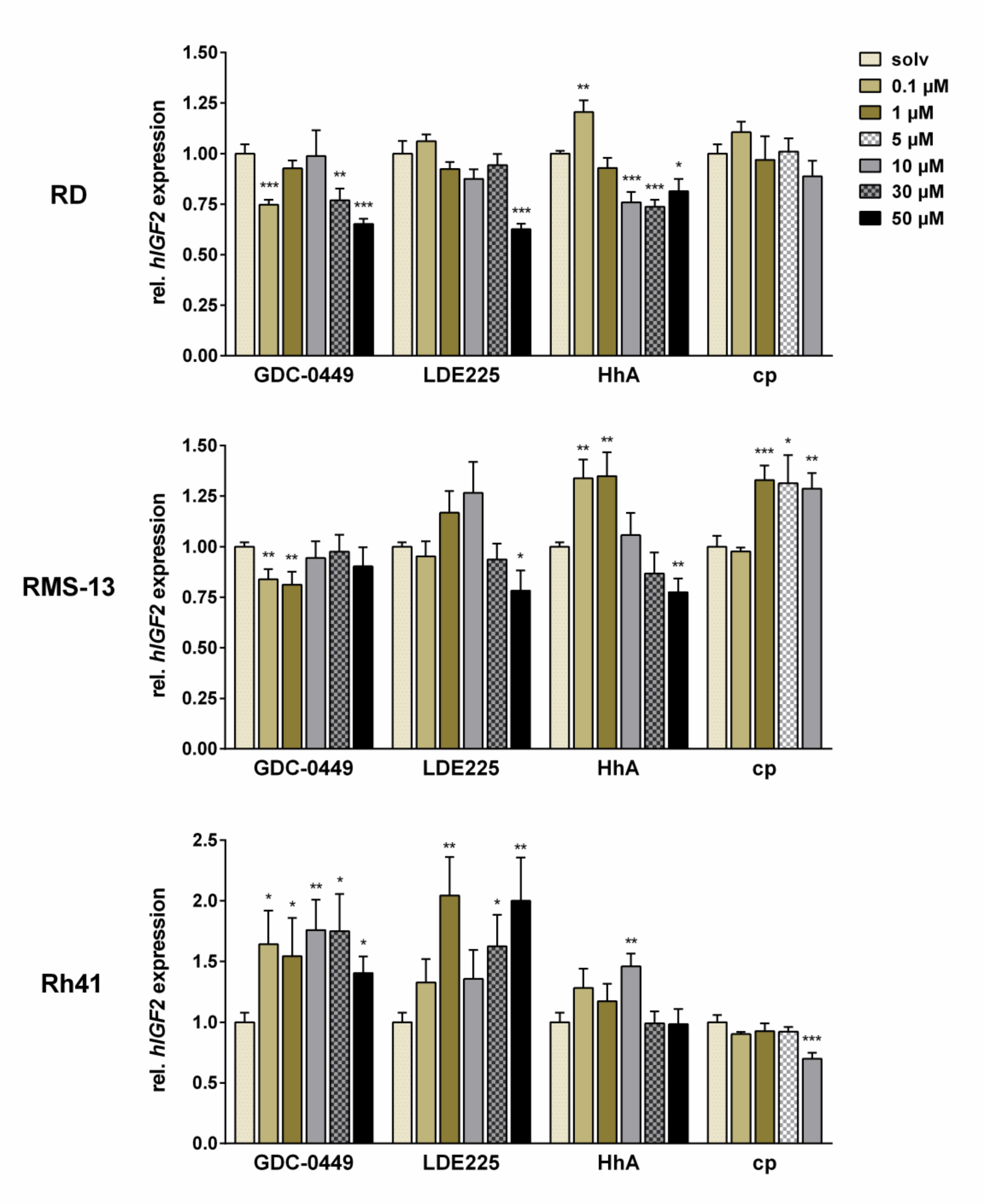

Supplemental Figure 3: Relative quantification of *MYOD* expression in RD, RMS-13 and Rh41 after treatment with GDC-0449, LDE225, HhA and cyclopamine (cp) at the concentration indicated. Gene expression levels were normalized to *18S* rRNA expression levels. The respective solvent-controls (solv) for each experiment were set to 1. All data represent at least 2 independent experiments performed in duplicates and measured in triplicates. The data are represented as mean +/-SEM; *, p<0.05; **, p<0.01; ***, p<0.001.

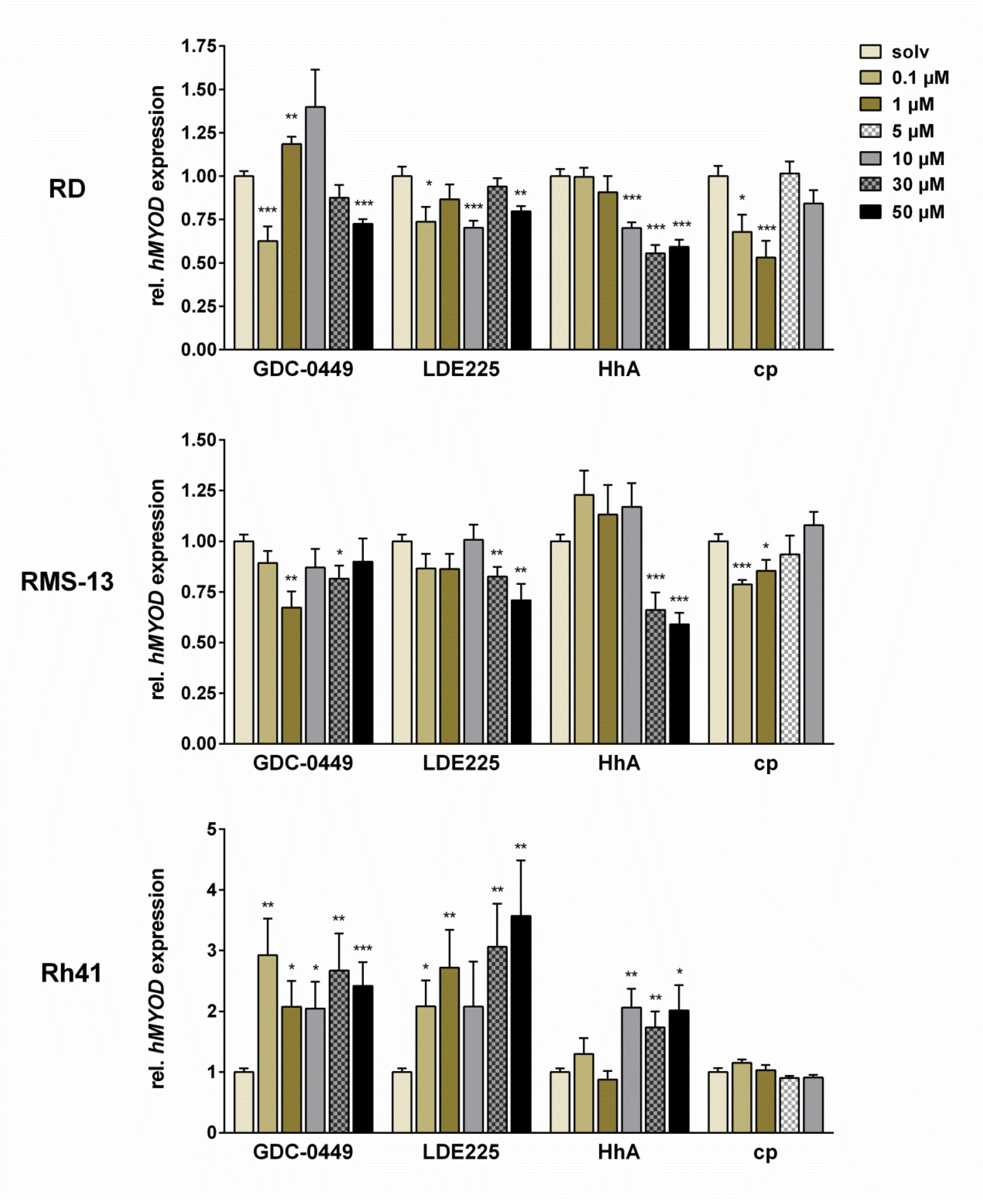

Supplemental Figure 4: Relative quantification of *MYH1* expression in RD, RMS-13 and Rh41 after treatment with GDC-0449, LDE225, HhA and cyclopamine (cp) at the concentration indicated. Gene expression levels were normalized to *18S* rRNA expression levels. The respective solvent-controls (solv) were set to 1. All data represent at least 2 independent experiments performed in duplicates and measured in triplicates. The data are represented as mean +/-SEM; *, $p<0.05$; **, $p<0.01$; ***, $p<0.001$.

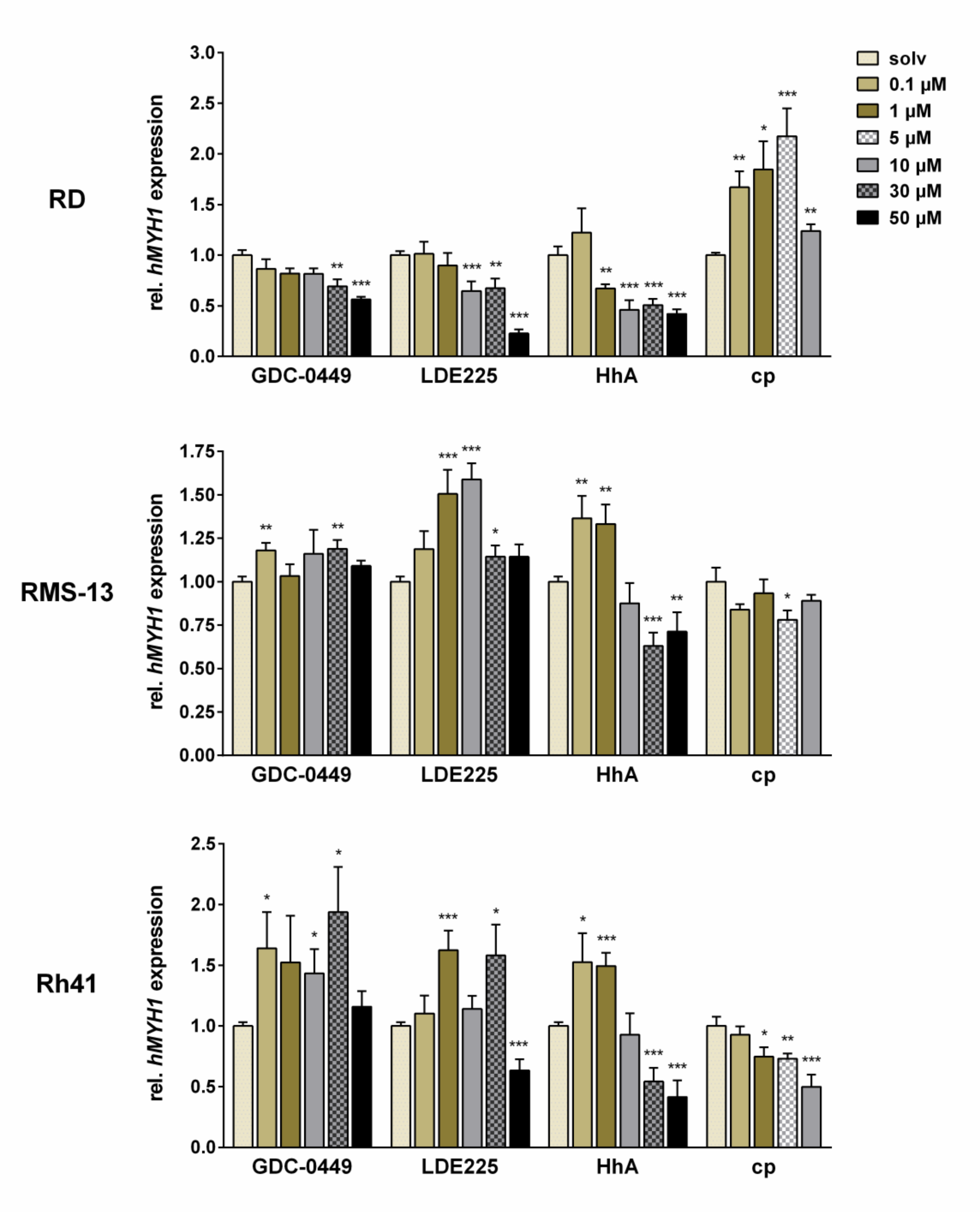

Supplemental Figure 5: Number of cells after treatment with GDC-0449, LDE225, HhA and cyclopamine (cp) at the indicated concentration. After treatment according to the experimental setup of BrdU incorporation assay, proliferation was estimated by counting of RD and Rh41 cells. The respective solvent-controls (solv) were set to 100 %. The data are represented as mean +/-SEM; *, $p<0.05$; **, $p<0.01$.

Although the decreases/increases in cell numbers follows the BrdU data (see Figure 2 in the main manuscript), the changes in cell numbers is not as pronounced. It is possible that this difference is due to a delayed cell division. Thus, the cell culture doubling time of Rh41 is 44 hours [7]. Therefore it is possible that 24 hours after treatment the cells already have incorporated BrdU (during s-phase), but have not yet divided (mitotic phase).

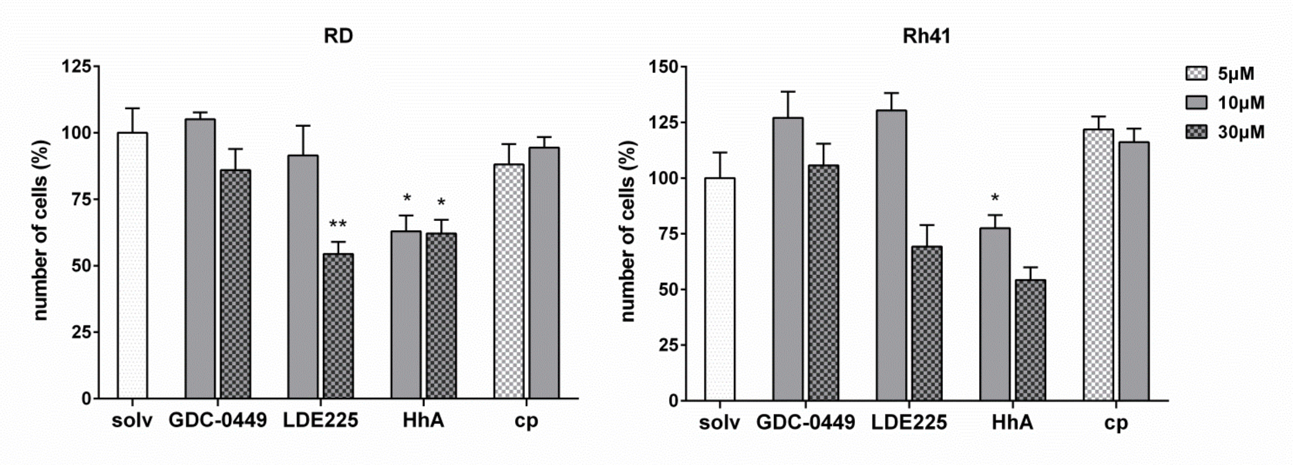

Supplemental Figure 6: Cell viability/metabolic activity of RD cells after treatment with GDC-0449, LDE225, HhA and cyclopamine (cp) at the indicated concentration as determined by WST assay. The respective solvent-controls (solv) were set to 100 %. The data are represented as mean +/-SEM; *, $p<0.05$; **, $p<0.01$.

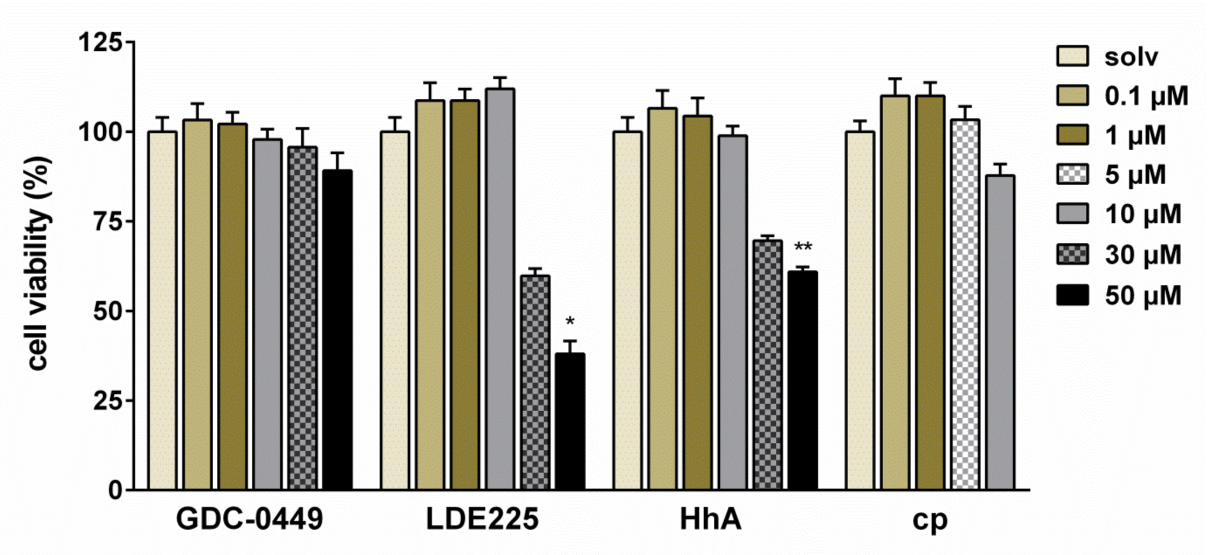

Supplemental references

1. Chen, J.K. *et al.* (2002) Inhibition of Hedgehog signaling by direct binding of cyclopamine to Smoothened. *Genes Dev*, **16**, 2743-2748.
2. Amakye, D. *et al.* (2013) Unraveling the therapeutic potential of the Hedgehog pathway in cancer. *Nat Med*, **19**, 1410-1422.
3. Ng, J.M. *et al.* (2011) The Hedgehog's tale: developing strategies for targeting cancer. *Nat Rev Cancer*, **11**, 493-501.
4. Rodon, J. *et al.* (2014) A phase I, multicenter, open-label, first-in-human, dose-escalation study of the oral smoothened inhibitor Sonidegib (LDE225) in patients with advanced solid tumors. *Clin Cancer Res*, **20**, 1900-1909.
5. Dijkgraaf, G.J. *et al.* (2011) Small molecule inhibition of GDC-0449 refractory smoothened mutants and downstream mechanisms of drug resistance. *Cancer research*, **71**, 435-444.
6. Yun, J.I. *et al*. (2012) Small Molecule Inhibitors of the Hedgehog Signaling Pathway for the Treatment of Cancer. *Arch Pharm Res*, **35**, 1317-1333.
7. Kang, M.H. *et al*. (2011) National Cancer Institute Pediatric Preclinical Testing Program: Model Description for In Vitro Cytotoxicity Testing. *Pediatr Blood Cancer*, **56**, 239-249.
